# Infectious spondylodiscitis: Epidemiology, diagnosis, microbiological findings, clinical features and outcomes in a 14-year retrospective study

**DOI:** 10.1007/s10096-025-05292-5

**Published:** 2025-09-29

**Authors:** Jacopo Conti, Nicholas Geremia, Stefano Di Bella, Fulvio Zadra, Verena Zerbato, Stella Babich, Venera Costantino, Manuela Di Santolo, Marina Busetti, Filippo Mearelli, Leonello Tacconi, Marco Francesco Maria Cavallaro, Maria Assunta Cova, Gianni Biolo, Roberto Luzzati, Donatella Giacomazzi

**Affiliations:** 1 AOU Policlinico di Modena, Università degli Studi di Modena e Reggio Emilia, Modena, 41121 Italy; 2 Unit of Infectious Diseases, Department of Clinical Medicine, Ospedale dell’Angelo, Venice, 30174 Italy; 3 Unit of Infectious Diseases, Department of Clinical Medicine, Ospedale Civile S.S. Giovanni e Paolo, Venice, 30122 Italy; 4 Clinical Department of Medical, Surgical, and Health Sciences, Trieste University, Strada di Fiume, 477, Trieste, 34129 TS Italy; 5 School of Medicine, Trieste University Hospital, Trieste, 34100 Italy; 6 Infectious Disease Unit, Trieste University Hospital, Azienda Sanitaria Universitaria Giuliano Isontina, Trieste, 34100 Italy; 7 Microbiology Unit, Trieste University Hospital, Azienda Sanitaria Universitaria Giuliano Isontina, Trieste, 34100 Italy; 8 Internal Medicine Unit, Trieste University Hospital, Azienda Sanitaria Universitaria Giuliano Isontina, Trieste, 34100 Italy; 9 Division of Neurosurgery, Trieste University Hospital, Azienda Sanitaria Universitaria Giuliano Isontina, Trieste, Italy; 10 Department of Radiology, Azienda Sanitaria Universitaria Giuliano Isontina, Ospedale Maggiore, Trieste, 34149 Italy; 11 Department of Radiology, Ospedale di Cattinara, University of Trieste, Strada di Fiume 447, Trieste, 34149 Italy; 12 Department of Medical Surgical and Health Sciences, Azienda Sanitaria Universitaria Giuliano Isontina, University of Trieste, Trieste, Italy; 13 Department of Medical Surgical and Health Science, Clinica Medica, Azienda Sanitaria Univesitaria Giuliano Isontina, University of Trieste, Trieste, 34127 Italy

**Keywords:** Infectious spondylodiscitis, Tuberculosis spondylodiscitis, Pyogenic spondylodiscitis, Osteomyelitis, CT-guided biopsy, Staphylococcus aureus

## Abstract

**Purpose:**

To describe the epidemiology, diagnosis, microbiological findings, clinical features, and outcomes of infectious spondylodiscitis (IS).

**Methods:**

Retrospective analysis of 98 IS patients (2010–2023 - Trieste Hospital). Clinical, radiological, and microbiological data analysed, multivariate logistic regression assessed risk factors for poor outcomes.

**Results:**

The incidence of IS was 3 cases per 100,000 inhabitants per year. Pyogenic infections accounted for 54% of cases, tuberculosis for 10%, while 35% remained of unknown etiology. Back pain (79%) and fever (72%) were the most common symptoms. *Staphylococcus aureus* was the most common pathogen, with 34 cases, representing 64% of pyogenic spondylodiscitis. Blood cultures were positive in 47% of pyogenic cases, 25% of all cases. PET/CT showed higher diagnostic utility (83%) than labeled leukocyte scintigraphy (54%). The lumbar spine was the most affected region (63%), followed by thoracic (25%) and cervical (12%). Neurosurgical biopsy showed a 33.3% positivity rate, while CT-guided biopsy yielded 22%. Cervical (OR 4.76) and thoracic (OR 3.89) involvement were associated with worse outcomes. Major complications included radicular nerve damage (51%), epidural/paravertebral abscesses (45%), endocarditis (14%), and a need for surgical intervention in 8%, with persistent neurological sequelae in 3%. Infection-related mortality rate was 6%.

**Conclusion:**

*S. aureus* remains the leading pathogen in IS. Blood cultures play a key role, yielding positive in half of pyogenic cases. PET/CT surpassed leukocyte scintigraphy and improved MRI diagnostic accuracy. Lumbar involvement correlates with better outcomes, and open biopsy provides a higher diagnostic yield than percutaneous TC-guided biopsy. Empirical therapy is required in one-third of cases.

## Introduction

Infectious spondylodiscitis (IS) is a severe condition characterized by spine inflammation affecting vertebral bodies and surrounding paraspinal structures. The disease can lead to significant complications such as abscess formation and vertebral destruction, which may result in neurological impairments [1]. Although IS is considered relatively uncommon, with an incidence of approximately 2.2 to 7.4 cases per 100.000 annually, its prevalence has gradually risen in recent years, nearly doubling in developed countries between 2010 and 2019 [2, 3]. Alarmingly, IS is associated with a significant mortality rate ranging from 3% to 20%, along with a decreased patient quality of life due to chronic pain and disability [4], underscoring the necessity for prompt diagnosis and effective management [5]. The increasing incidence of IS, driven in part by an aging population and the rising prevalence of chronic diseases [6, 7], highlights the importance of early and accurate diagnosis, as well as the need for tailored treatment strategies.

The two most common forms of IS are pyogenic spondylodiscitis (PS) and tuberculous spondylodiscitis (TS). PS constitutes 40% to 80% of all IS cases and is predominantly associated with pyogenic bacteria, with *Staphylococcus aureus* being the most frequent pathogen. In contrast, TS, which constitutes 17% to 40% of cases, is recognized as one of the most severe extrapulmonary manifestations of tuberculosis, particularly in developing countries [8, 9]. Despite advancements in medical imaging and laboratory diagnostics, the differentiation between PS and TS remains challenging due to overlapping clinical presentations, often leading to delayed diagnosis and treatment, both of which are independent risk factors for poor outcomes [10–12].

The diagnostic process for spondylodiscitis typically involves a combination of clinical, radiological, and microbiological assessments. Magnetic resonance imaging (MRI) is considered the gold standard for diagnosing spinal infections, displaying significant sensitivity (92%) and specificity (96%) [13]. However, the time frame from symptom onset to conclusive diagnosis is often delayed, ranging from 35 to 50 days [14], contributing to the complexity of managing the condition.

Further complicating treatment is the variability in causative pathogens and their antibiotic resistance patterns, which differ significantly across different regions worldwide [15]. Identifying the underlying pathogen is fundamental for initiating targeted and effective antibiotic therapy. In cases where blood cultures or serological tests fail to confirm a known organism (e.g., *S. aureus*,* Staphylococcus lugdunensis*,* Brucella*), image-guided biopsy is recommended for diagnosis, as highlighted by clinical guidelines [16–18].

The distinct anatomical characteristics of spinal infections necessitate effective antibiotic therapy with adequate bone penetrations; however, surgical intervention may be indicated in cases of progressive neurological deficits, spinal instability, spinal abscess formation, persistent bloodstream infection without an alternative source or when conservative management proves inadequate [19].

This study aims to characterize the causative pathogens associated with spinal infections in our institution, evaluating complications and outcomes through a comprehensive analysis of clinical, radiological, and microbiological data.

## Materials and methods

We conducted a retrospective, single-center study at the University Hospitals of Trieste, collecting all the consecutive cases of IS between January 1, 2010, and December 31, 2023.

These healthcare institutions act as the primary center for tertiary medical services, located in an urban area in northeastern Italy, serving a population of roughly 230,000 inhabitants [20].

### Study objectives

This study aimed to describe the epidemiology, diagnostic approaches, microbiological findings, clinical characteristics, and outcomes of infectious spondylodiscitis (IS) in patients treated at the University Hospitals of Trieste over a 14-year period (2010–2023).

### Inclusion/exclusion criteria and definitions

Diagnosis of IS was suspected in the case of back pain unrelieved by rest or analgesics, with or without fever and/or neurological deficits. Altered inflammation markers, such as leukocytosis and increased values of C-reactive protein, may also be present. Diagnosis of IS was performed through imaging studies, such as MRI, positron emission tomography/computed tomography (PET/CT) or scintigraphy scan, which confirmed the clinical suspicion of vertebral and intervertebral disc abnormalities. The etiological diagnosis of spondylodiscitis was established by (i) culture of CT-guided percutaneous needle aspiration/biopsy, or (ii) open neurosurgical vertebral or paravertebral and vertebral biopsy, or (iii) blood cultures, requiring at least two positive cultures for potential contaminant microorganisms (e.g., *Staphylococcus epidermidis*) and one positive culture for other pathogens.

When available, histological examination also aided in diagnosis (e.g., caseating granuloma for tuberculosis). In the absence of microbiological or histological confirmation, patients presenting with clinical-radiological characteristics suggestive of spondylodiscitis who showed clinical improvement with empiricalantibiotic therapy were also included in our study. All patients under 18 years of age, those with prior spinal surgery, or individuals with recent spinal trauma were excluded.

Neurological symptoms were defined as the presence of radicular pain, reflex abnormalities, sensory-motor deficits, and paralysis.

Appropriate antibiotic therapy was defined as an active treatment against the microorganism isolated based on antimicrobial susceptibility testing (AST). For unknown etiology IS, appropriate antibiotic therapy was based on empiric antibiotic therapy according to IS guidelines [21].

The clinical outcomes assessed in this study included: (i) infection-related death, (ii) complete recovery, (iii) relapse or recurrence of infection, and (iv) the necessity of surgical source control. Unfavorable outcomes were defined as any of the following: infection-related mortality, relapse or recurrence during or after appropriate antibiotic therapy, persistent neurological deficits at the end of follow-up, incomplete symptom resolution, or the indication for neurosurgical intervention to manage the source of infection.

Follow-up assessments were primarily conducted through outpatient visits. Additionally, a single telephone follow-up assessment was conducted in May 2024. During this interview, the persistence of neurological symptoms and/or disabling spinal pain was evaluated, with the latter assessed using the ‘Oswestry Low Back Disability Questionnaire’ [22, 23].

### Microbiological diagnosis

Microbiological diagnosis was performed using culture-based and molecular methods to optimize pathogen identification. Blood cultures, at least two samples, were systematically collected before antimicrobial therapy initiation and incubated in automated systems (Bact/Alert^®^ Virtuo^®^, bioMérieux) for at least five days to maximize detection sensitivity.

For direct sampling, specimens were obtained through CT-guided percutaneous biopsy, fine-needle aspiration, or open surgical biopsy. Samples were inoculated on aerobic and anaerobic culture media, including blood agar (COS), chocolate agar (PVX), MacConkey agar (MCK), Sabouraud Dextrose (SGC2) Agar as well as thioglycolate broth (THIO-T), and incubated for up to 14 days to detect slow-growing pathogens. Bacterial identification was performed by MALDI-TOF mass spectrometry (bioMérieux) and susceptibility tests by the Vitek2 system (bioMérieux). For fastidious organisms and anaerobes, we used the disc diffusion method (Liofilchem^®^). Antimicrobial susceptibility testing was conducted with (Beckman Coulter Diagnostics). Susceptibility results were interpreted according to EUCAST criteria.

To enhance pathogen detection, the FilmArray BioFire^®^ Joint Infection Panel (BF-JIP, bioMérieux, Marcy-l’Étoile, France) multiplex polymerase chain reaction (PCR) assay was applied directly to biopsy specimens, facilitating the identification of common bacterial and fungal pathogens, including antimicrobial resistance genes [24]. This assay was introduced in our microbiology laboratory at the end of 2022 and was employed from that time until the conclusion of the study. Consequently, the number of samples analyzed using this technique was limited.

Ziehl-Neelsen staining was performed for suspected TS, and mycobacterial culture was conducted using solid Löwenstein-Jensen medium and the Mycobacteria Growth Indicator Tube liquid culture system. Additionally, PCR targeting *Mycobacterium tuberculosis* complex was performed to improve diagnostic sensitivity.

### Ethics

The local Ethics Committee (approval number 112_2020) in accordance with the Declaration of Helsinki (1964) and its subsequent amendments approved this retrospective study. As the study design is retrospective, the requirement for written informed consent was waived.

### Data collection and statistical analysis

A comprehensive review of medical records was conducted, focusing on demographic data, potential predisposing factors or comorbidities, signs and symptoms and diagnostic delay between symptom onset and the time of instrumental diagnosis. Additionally, spinal involvement with vertebral extension, microbiological and histological results, therapeutic management, duration of follow-up, outcome at the end of hospitalization and follow-up, as well as any recurrence, were examined. The vertebral involvement was coded as a binary variable for each specific spinal segment affected.

Datawere collected using Microsoft Excel (Redmond, WA, USA). Quantitative variables were described using the median and interquartile range (IQR), while qualitative variables were summarized with absolute and relative frequencies (percentage). Continuous variables were compared using the Kruskal-Wallis test and categorical variables were evaluated using either the Chi-squared or Fisher’s exact test, as appropriate. Univariate logistic regression was employed to assess risk factors associated with unfavorable outcomes. A stepwise approach was utilized to multivariate logistic regression to determine the same parameters. A confidence interval (CI) of 95% was considered and a p-value (p) ≤ 0.05 was deemed statistically significant. Statistical analyses were performed with StataNow BE version 18.5 (StatsCorp, Lakeway, TX, USA).

## Results

**Fig. 1:**
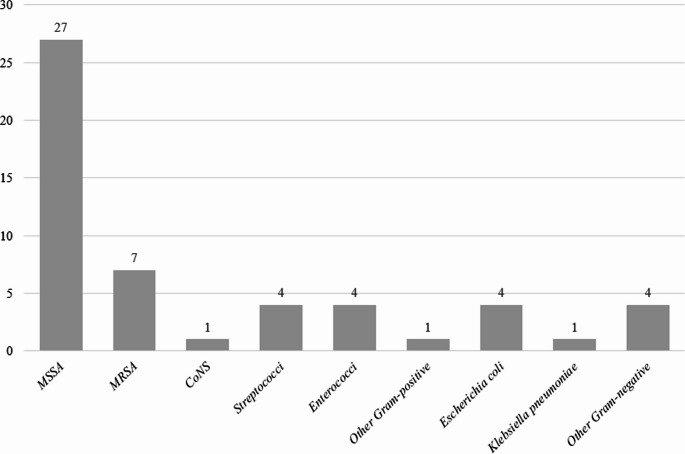
Distribution of bacterial isolates identified in pyogenic spondylodiscitis clinical samples. CoNS: Coagulase-Negative Staphylococci; MRSA: methicillin-resistant S. aureus; MSSA: methicillin-susceptible S. aureus

Nineteen-eight patients were included with the diagnosis of IS during the study period from 2010 to 2023, resulting in an incidence rate of 3.04 cases per 100.000 inhabitants per year. The overall incidence rate for PS was 1.65 per 100.000 inhabitants per year; instead, the incidence rate for TS was 0.31 per 100,000 inhabitants per year. Fifty-three (54.1%) cases of IS were caused by pyogenic bacteria, 10 cases (10.2%) by *M. tuberculosis* and 35 cases (35.7%) had unknown etiological causes. Most cases of PS were related to *S. aureus* with 27 (51%) being methicillin-susceptible *S. aureus* (MSSA) and 7 (13.2%) being methicillin-resistant *S. aureus* (MRSA). Other microbiological microorganisms isolated were reported in Fig. 1. Twenty-five cases (47.2%) had positive blood cultures for PS. When considering the entire cohort, blood cultures were positive in 25.5% of all cases.

Among IS cases, 22 patients (22.5%) underwent neurosurgical open biopsy, with positive microbiological results in 7 cases (33.3%). CT-guided needle aspirates were performed in 18 IS patients (18.4%), yielding positive microbiological results in 4 cases (22.2%).

The median age of the patients was 71.0 (IQR 57.0–80.0) years old. The median age of the TS group was lower (59.5 years, IQR 35.0–78.0) than that of the PS group (71.0 years, IQR 62.0–81.0) and the undiagnosed group (74.0 years, IQR 59.0–79.0). Most of the included patients were male (68, 69.4%). The majority of PS and IS of unknown etiology occurred in Italian natives, specifically, 51 out of 53 PS cases (96.2%) and 33 out of 35 cases of IS with unknown etiology (94.3%) were diagnosed in this population.

Back pain was the most frequent symptom (78 cases, 79.6%), followed by fever (71 cases, 72.5%). The demographic, clinical characteristics, and infection origin data are presented in Table 1.

**Table 1:** Demographic, clinical characteristics, and infection origin in patients with spondylodiscitis, stratified by etiology

	Pyogenic (53)	Tuberculosis (10)	Unknown etiology (35)	*p*-value
Age, median (IQR)	71.0 (62.0–81.0)	59.5 (35.0–78.0)	74.0 (59.0–79.0)	0.40
Male, n.° (%)	39 (73.6)	8 (80.0)	21 (60.0)	0.30
Origin				
Italy, n.° (%)	51 (96.2)	6 (60.0)	33 (94.3)	**< 0.0001**
European, n.° (%)	1 (1.9)	0 (0.0)	1 (2.9)	
Asian, n.° (%)	0 (0.0)	2 (20.0)	0 (0.0)	
African, n.° (%)	0 (0.0)	2 (20.0)	0 (0.0)	
Latin American, n.° (%)	1 (1.9)	0 (0.0)	0 (0.0)	
Unknown, n.° (%)	0 (0.0)	0 (0.0)	1 (2.9)	
Comorbidity				
Diabetus mellitus, n.° (%)	14 (26.4)	3 (30.0)	7 (20.0)	0.74
Solid tumor, n.° (%)	11 (20.8)	2 (20.0)	5 (14.3)	0.74
Haematological disease, n.° (%)	11 (20.8)	2 (20.0)	3 (8.6)	0.27
Hepatic disease, n.° (%)	8 (15.1)	2 (20.0)	4 (11.4)	0.70
Chronic renal disease, n.° (%)	3 (5.7)	2 (20.0)	4 (11.4)	0.24
Autoimmune disease, n.° (%)	2 (3.8)	0 (0.0)	0 (0.0)	0.61
PWID, n.° (%)	5 (9.4)	1 (10.0)	2 (5.7)	0.76
Symptoms				
Back Pain, n.° (%)	37 (69.8)	9 (90.0)	31 (88.6)	0.07
Fever, n.° (%)	38 (71.7)	9 (90.0)	23 (65.7)	0.32
Neurological signs, n.° (5)	7 (13.2)	2 (20.0)	5 (14.3)	0.76
Primary focus of infection				
Primary origin	28 (52.8)	0 (0.0)	16 (45.7)	**0.004**
Unknown origin, n.° (%)	18 (34.0)	10 (100.0)	14 (40.0)	
Bacteriemic origin, n.° (%)	7 (13.2)	0 (0.0)	5 (14.3)	

**Fig. 2:**
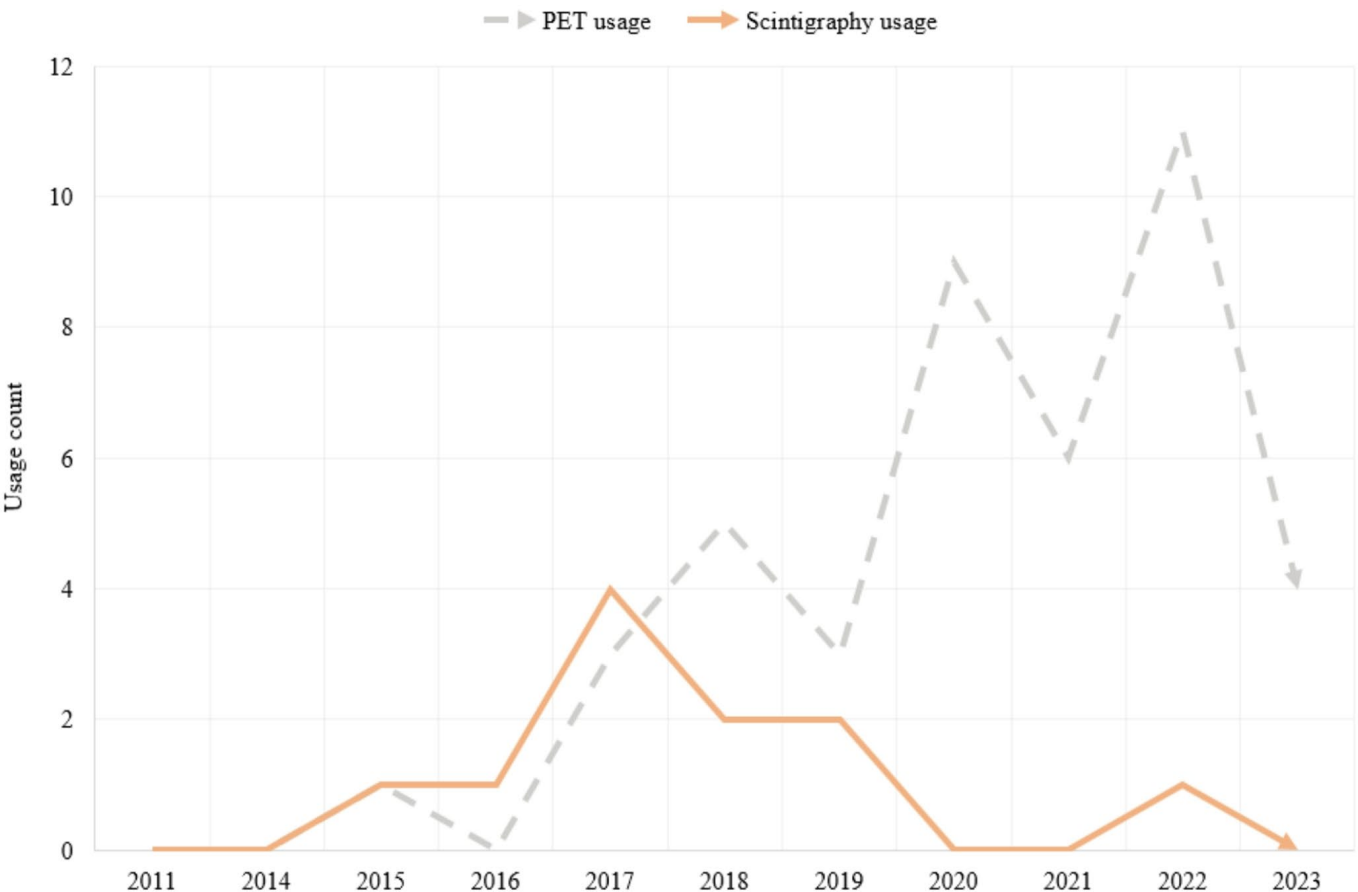
PET and scintigraphy yearly rates

Patients with suggestive clinical symptoms but inconclusive or negative first-level imaging MRI required further investigation for confirmation using nuclear medicine techniques. Forty-seven patients underwent second-level imaging, including 42 PET/CT (35/42 were positive, 83.3%) and 11 scintigraphy scans (6/11 were positive, 54.5%). PET/CT has progressively replaced scintigraphy in the diagnosis of IS in our study. The timeline of second-level nuclear medicine techniques performed is reported in Fig. 2.

The most frequently observed complications in IS were radicular nerve damage and abscess formation. Radicular nerve damage occurred in 35 cases (66.0%) of PS and 5 cases (50.0%) of TS. Muscular or epidural abscesses were observed in 44 out of 98 patients (44.9%) at almost similar rates across the three groups, affecting 24 patients (45.3%) in PS, 5 patients (50.0%) in TS, and 15 patients (42.9%) in IS of unknown etiology. The occurrence of endocarditis was not uncommon in our cohort, occurring in 14.3% of IS cases. Microbiological findings, complications, treatment, and outcomes in patients with spondylodiscitis by etiology are presented in Table 2.

**Table 2:** Microbiological findings, complications, treatment, and outcomes in patients with spondylodiscitis by etiology

	Pyogenic (53)	Tuberculosis (10)	Unknown etiology (35)	*p*-value
Positive blood culture, n.° (%)	25 (47.2)	/	/	
Complications				
Radicular nerve damage, n.° (%)	35 (66.0)	5 (50.0)	10 (28.6)	**0.003**
Muscular or epidural abscess, n.° (%)	24 (45.3)	5 (50.0)	15 (42.9)	0.92
Endocarditis, n.° (%)	8 (15.1)	1 (10.0)	5 (14.3)	1.00
Skin localizations, n.° (%)	5 (9.4)	3 (30.0)	0 (0.0)	**0.009**
Vertebral involvement				
Cervical region, n.° (%)	6 (11.3)	0 (0.0)	6 (17.1)	0.39
Thoracic region, n.° (%)	15 (28.3)	1 (10.0)	9 (25.7)	0.59
Lumbar region, n.° (%)	29 (73.6)	9 (90.0)	24 (68.6)	0.40
Sacral region, n.° (%)	10 (18.9)	2 (20.0)	6 (17.1)	1.00
> 2 vertebrae involved, n.° (%)	14 (24.5)	0 (0.0)	6 (17.1)	0.22
Diagnostic delay (days), median (IQR)	23.0 (7.0–30.0)	35 (16.0–60.0)	40 (17.0–60.0)	**0.02**
Hospitalization (days), median (IQR)	33.0 (21.0–59.0)	21.0 (14.0–35.0)	25.0 (12.0–49.0)	0.24
Antibiotic therapy				
Antibiotics 30 days prior, n.° (%)	19 (35.9)	1 (10.0)	8 (22.9)	0.16
Combination-therapy, n.° (%)	29/41 (70.7)	/	20/28 (71.4)	1.00
Antibiotic duration (days), median (IQR)	90.0 (6.0–120.0.0.0)	360.0 (270.0–360.0.0.0)	60.0 (0.0–90.0)	**0.0001**
Unfavourable outcomes				
Infection-related death, n. (%)	5 (9.4)	0 (0.0)	1 (2.9)	0.46
Recovery, n.° (%)	45 (84.9)	6 (60.0)	28 (80.0)	0.19
Relapse, n.° (%)	5 (9.4)	0 (0.0)	2 (5.7)	0.74
Necessity of surgical source control, n.° (%)	3 (5.7)	0 (0.0)	5 (14.3)	0.29

**Fig. 3:**
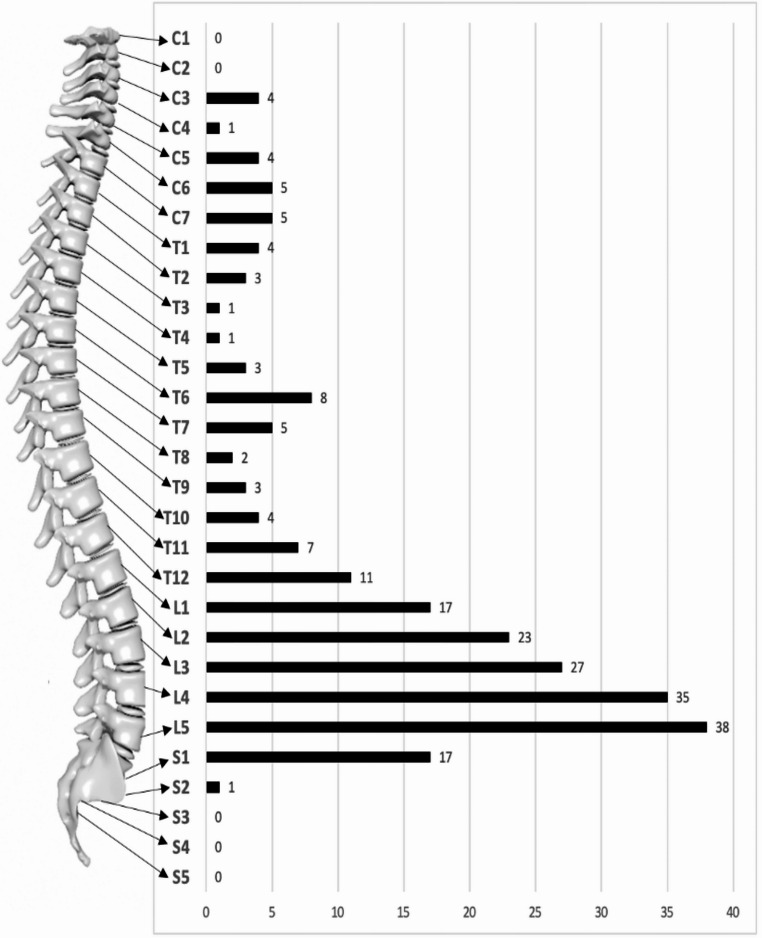
Vertebral distribution of infectious spondylodiscitis cases, illustrating the frequency of involvement across cervical, thoracic, lumbar, and sacral regions

Cervical and thoracic localization were observed in 12 (12.2%) and 25 (25.5%) cases, respectively. Lumbar IS (62 cases, 63.2%) was the most frequent vertebral region involved, and the most affected segment was L5 (38 cases, 61.3%). In 20 cases (20.4%), spine involvement was observed in more than two contiguous vertebrae and their corresponding intervertebral disc. Figure 3 shows the different vertebral localizations.

The diagnostic delay was found to be different among PS, ST, and unknown etiology cases; the latter group had the longest period from the onset of symptoms to diagnosis, with a median of 40 days (IQ 17.0–60.0).

In the analyzed cohort of IS patients, the following unfavorable outcomes were observed: infection-related deaths occurred in 6 cases (6.1%), including 5 cases of PS and 1 case of IS of unknown origin; recurrences were reported in 7 cases (7.1%), with 5 occurring in PS and 2 in IS of unknown etiology; and 8 (8.2%) patients required surgical debridement, including 3 with PS and 5 with IS of unknown etiology.

Statistical analysis revealed that involvement of the cervical region [OR 4.76 (1.10–21.39), *p* = 0.04] and the thoracic region [OR 3.89 (1.12–13.52), *p* = 0.03] were associated with an increased risk of worse outcomes compared to lumbar region involvement. The multivariate analysis is reported in Table 3.

**Table 3:** Univariate and multivariate logistic regression of factors associated with unfavourable outcomes

	OR (95% IC)	*p*-value	OR (95% IC)	*p*-value
Age	1.03 (0.99–1.07)	0.19	1.02 (0.98–1.06)	0.35
Male sex	0.53 (0.19–1.49)	0.23	0.77 (0.23–2.56)	0.67
Etiology				
Unknown	Ref	Ref	Ref	Ref
Pyogenic	1.17 (0.41–3.34)	0.77	1.26 (0.37–4.22)	0.71
Diagnostic delay	1.00 (0.98–1.01)	0.50	1.00 (0.98–1.01)	0.57
Comorbidities				
Diabetus Mellitus	0.52 (0.14–1.96)	0.33		
Solid tumor	1.81 (0.55–5.91)	0.32		
Haematological disease	0.95 (0.24–3.74)	0.94		
Hepatic disease	1.84 (0.51–6.66)	0.35		
PWID	2.78 (0.60–12.8)	0.19	5.10 (0.87–29.9)	0.07
Complications				
Endocarditis	1.17 (0.29–4.64)	0.83		
Radicular nerve damage	1.41 (0.51–3.88)	0.51		
Muscular or epidural abscess	1.92 (0.70–5.29)	0.21		
Vertebral involvement				
Cervical region	3.67 (1.02–13.25)	0.05	4.76 (1.10–21.39.10.39)	0.04
Thoracic region	2.65 (0.92–7.63)	0.07	3.89 (1.12–13.52)	0.03
Lumbar region	0.41 (0.14–1.16)	0.09		
Sacral region	1.24 (0.36–4.30)	0.74		
> 2 vertebrae involved	2.34 (0.75–7.29)	0.14	2.31 (0.67–8.00.67.00)	0.18

Follow-up assessments through outpatient visits had a mean duration of 112 days for PS (range 84–141), 322 days for TS (range 222–416), and 122 days for IS of unknown origin (range 93–163). Telephone follow-up assessment was conducted for 54.1% of IS patients, with a mean follow-up duration of 4 years and 4 months, ranging from 6 months to 13 years. Among the 53 patients who completed the Oswestry Disability Index assessment, 35 (66%) achieved complete recovery (score 0–4), while 18 (34%) continued to experience pain-related disability (score > 5). Among these, three patients exhibited persistent neurological symptoms, including bilateral lower limb paresthesia (2 cases) and paraplegia (1 case). Recovery rates were highest in the TS group (88%), compared to PS (63%) and IS of unknown origin (62%). Symptom persistence was more frequent in pyogenic cases, with rates similar to those of unknown origin, compared to tuberculosis-related cases.

## Discussion

IS has been considered a relatively uncommon disease, whose diagnosis resulted to be particularly challenging [25]. The worldwide incidence is estimated to range from 2.2 to 7.4 cases per 100.000 inhabitants per year [2, 3, 26]. Despite being an uncommon condition, IS has been diagnosed with increasing frequency in recent years. Several authors have reported a rise in its incidence in the past few years, attributed to improved diagnostic tests, the resurgence of tuberculosis, the increase in the numbers of older patients with chronic diseases or immunocompromised states, as well as the rise in bacteremia and iatrogenic infections [26, 27]. In a recent article, Song et al. reported a 1.5-fold increase in the incidence of PS in South Korea during the observational period from 2010 to 2019, with rates changing from 22.90 to 35.79 per 100,000 inhabitants per year [27]. This epidemiological change has been influenced by the increasing number of frail individuals, particularly older adults with multiple comorbidities and compromised immunity.

Our study revealed an overall incidence rate of 3 cases per 100,000 inhabitants per year, aligning with previously reported data in the literature [2]. Notably, the incidence of pyogenic spondylodiscitis alone was 1.65 per 100,000 inhabitants per year, consistent with available epidemiological findings.

MRI remains the gold standard for diagnosing spondylodiscitis, particularly when enhanced with contrast media [28]. Our study highlighted that patients presenting with a negative or inconclusive MRI results, yet exhibiting clinical symptoms suggestive of spinal pathology, required second-level imaging. Among the imaging techniques employed, PET/CT was the most effective, corroborating existing literature data [29]. PET/CT improved or refined the radiological diagnosis in 83.3% of cases, compared to only 54.5% of bone scintigraphy cases. Although the advent of new diagnostic techniques has improved the diagnosis of IS, a significant diagnostic delay persists.

Our findings support that back pain and fever are the most common clinical manifestations of spondylodiscitis, aligning with the established clinical presentation of the disease and consistent with previously reported literature. Among the three main symptoms, neurological signs were the least frequent.

In line with results from other studies [27, 30–33], microbiological diagnosis was achieved in approximately two-thirds of our cases (64.3%). Consequently, a relatively high number of patients required empirical antibiotic treatment. This finding suggests an inherent challenge in managing IS without microbiological confirmation. Clinicians are frequently required to initiate antibiotic therapy to prevent disease progression, yet microbiological data are often lacking at the time treatment decisions must be made to reduce the risk of complications. The least invasive, least costly, and most sensitive methods for microbiological confirmation are blood cultures, which identified the microorganism in 25.5% of all IS cases and in 47.2% of cases among patients with PS in our cohort. The low sensibility of blood culture may significantly impact microbiology diagnostics; our data did not notably differ from findings in the international literature which reports a prevalence ranging from 25% to 59% [13].

According to this study, cultures obtained during surgical interventions or open biopsy yielded poor results (33.3%) compared to those reported in the literature [34, 35], and similar findings were observed for CT-guided needle aspirates (22.2%). A potential explanation for these findings is that a significant proportion of patients had undergone empirical antibiotic therapy in the 30 days preceding the biopsy, which may have reduced bacterial load and, consequently, culture positivity. Specifically, pre-biopsy antibiotic exposure was documented in 35.9% of patients with pyogenic spondylodiscitis, 10.0% of those with tuberculous spondylodiscitis, and 22.9% of cases with an unknown etiology, further supporting the hypothesis that prior antimicrobial treatment negatively impacted microbiological diagnosis. Moreover, only a limited number of cases underwent “fast microbiology” techniques, which could have further contributed to the low overall diagnostic yield.

In our cohort, the median duration from the onset of symptoms to diagnosis differed across the three groups studied. The group with unknown etiology IS experienced the longest diagnostic delay, averaging 40.0 days (IQR 17.0–60.0), compared to the TS and PS groups. However, diagnostic delay was not statistically associated with an unfavorable outcome in the logistic regression analysis. This unexpected result may be due to the relatively low number of cases enrolled in this study.

The average duration of antibiotic therapy was found to be quite different among the three etiological groups. Of course, tuberculosis requires long-term antibiotic therapy. On the other hand, the PS group had a median antibiotic treatment duration of 90 days (IQR 6.0–120.0.0.0), while the therapy for unknown etiology IS lasted a median of 60.0 days (IQR 0.0–90.0). This difference might be due to a lower percentage of patients receiving antibiotics prior to etiological diagnosis. According to the Infectious Diseases Society of America guidelines, antibiotic therapy for non-tuberculous uncomplicated bacterial osteomyelitis should generally not exceed 6 weeks; however, it may extend up to three months for *Brucella* infections [16]. The longer duration observed in our study may be partially explained by including patients from 2010 onward, preceding the publication of current guidelines and more recent studies that support shorter treatment regimens [36]. Additionally, our cohort predominantly consisted of elderly adults who complained with frailty factors and comorbidities which likely contributed to the need for a more intensive and prolonged therapeutic approach [37].

Regarding outcomes, localization and etiology of IS can be crucial points in determining the risk of disability sequelae. In our multivariate logistic regression analysis, cervical and thoracic localizations were associated with the worst outcomes; this aligns with anatomical and biomechanical considerations, as these regions have a higher risk of instability and neurological complications. The distribution of IS varies by etiology: PS predominantly affects the lumbosacral region, while TS traditionally involves the thoracolumbar transition zone, in accordance with previous literature [38]. Tuberculosis is more commonly associated with spinal deformities, epidural and paravertebral abscesses, and neurological impairment due to the destructive nature of caseating granulomas [39]. Although etiology is recognized as an important determinant of disease burden, in our cohort, it was not statistically associated with an unfavorable outcome.

Surgical intervention is indicated in cases involving spinal cord or nerve root compression, instability, vertebral deformities, pathological fractures, or abscess drainage [39, 40]. In our study, surgery was primarily performed for spinal cord decompression and stabilization, with a higher frequency observed in cases of unknown etiology and PS. This finding contrasts with recent studies that emphasize thoracic spondylitis as the primary indication for surgical intervention [31, 41, 42].

At follow-up, symptom persistence was more frequent in PS and cases of unknown origin compared to TS. This may be partially explained by the younger age of TS patients and their longer follow-up duration. Our findings are consistent with previous research. Bhagat et al. [17] reported that patients with culture-negative spondylodiscitis had comparable or even worse outcomes than those with culture-positive infections, highlighting the challenge of managing cases without microbiological confirmation. Similarly, Colmenero et al. [41] found that severe functional sequelae were observed in 32.3% of patients at six months, a rate slightly lower but comparable to our study. Additionally, their comparative study reported spinal disability, including persistent pain and neurological deficits, in 15.4% of patients with culture-negative spondylodiscitis and 20.5% of those with pyogenic etiology, values lower than our findings (36% and 40%, respectively).

Our findings align with previous research. Bhagat et al. [17] reported that patients with culture-negative spondylodiscitis had outcomes comparable to or worse than those with culture-positive infections, emphasizing the challenges in managing cases without microbiological confirmation. Similarly, Colmenero et al. [41] found that severe functional sequelae were present in 32.3% of patients at six months, a slightly lower but comparable rate to our study. Furthermore, their comparative analysis reported spinal disability, including persistent pain and neurological deficits, in 15.4% of patients with culture-negative spondylodiscitis and 20.5% of those with pyogenic etiology, both lower than the rates observed in our cohort (36% and 40%, respectively).

This study presents several limitations that should be considered when interpreting the findings. Firstly, the relatively small sample size may affect the statistical power and generalizability of the results. Additionally, the retrospective and single-center design limits the external validity of the study, as the findings may not be applicable to different settings or populations. The prolonged enrollment period, spanning 14 years, introduces the potential for variations in medical practices and treatment protocols over time. Furthermore, there was heterogeneity in the duration and selection of antibiotic therapies, which could influence the comparability of treatment outcomes across the study population.

Despite these limitations, the study also possesses notable strengths. A rigorous case definition and outcome assessment were employed, ensuring consistency and reliability in data interpretation. The diagnostic approach was homogeneous, including the use of cultures, fine-needle aspiration biopsies, and/or open biopsies, thereby enhancing pathogen identification accuracy. Importantly, the study reports a favorable clinical outcome, with complete recovery or improvement in most cases, highlighting the overall effectiveness of the management strategies implemented.

## Conclusions

This study underscores the significance of IS as a clinically pertinent condition within the studied cohort, frequently marked by diagnostic complexity and delays attributable to its nonspecific clinical manifestations. Despite advancements in microbiological diagnostics, a substantial proportion of cases remain without an etiological diagnosis, necessitating empirical antibiotic therapy in nearly one-third of patients.

*S. aureus* remains the predominant causative pathogen, reinforcing the need for early targeted antimicrobial strategies. Blood cultures play a fundamental role in microbiological confirmation, yielding positive results in almost half of pyogenic cases.

Our findings further support the growing role of PET/CT, which demonstrated superior diagnostic accuracy compared to labeled leukocyte scintigraphy and proved valuable in complementing MRI for early detection of doubtful cases. The anatomical localization of the infection emerged as a key prognostic factor, with lumbar involvement being associated with more favorable clinical outcomes, whereas cervical and thoracic infections were linked to a higher risk of complications.

Additionally, open biopsy provided a higher microbiological diagnostic yield than percutaneous techniques, underscoring its importance in cases where noninvasive methods fail to identify the causative agent.

In conclusion, timely and multidisciplinary management is essential to optimize patient outcomes, minimize complications, and reduce long-term sequelae.

## Data Availability

No datasets were generated or analysed during the current study.
